# Deinking of Post-Consumer Waste Flakes—Objective Assessment of Ink Removal on Inhomogeneous Film Fractions

**DOI:** 10.3390/polym18060765

**Published:** 2026-03-21

**Authors:** Steven Zimmer, Lukas Seifert, Rainer Dahlmann

**Affiliations:** Institute for Plastics Processing (IKV) in Industry and Craft, RWTH Aachen University, Seffenter Weg 201, 52074 Aachen, Germany; steven.zimmer@ikv.rwth-aachen.de (S.Z.); lukas.seifert@ikv.rwth-aachen.de (L.S.)

**Keywords:** polymer recycling, circular economy, waste management, deinking, statistical analysis

## Abstract

The deinking of plastic packaging waste offers the potential of decreasing contamination and thus increasing the overall quality of recycled plastics, enabling their use in more demanding applications. However, for flexible polyethylene packaging waste, deinking is not yet implemented on an industrial scale and there is currently no objective methodology to evaluate the deinking effect on those inhomogeneous flakes. In this study, a novel approach for the objective assessment of ink removal on flexible post-consumer waste (PCW) is proposed. Via an image-based analysis, the transparency of the flakes is transformed into the 8-bit grey scale, and the deinking efficiency of several experiments is compared via the skewness and median of grey value distributions. The method is compared to the International Commission on Illumination (CIE) Lab-method and its robustness against wrinkles and overlaps is critically discussed. Using this analysis method enables the investigation of the general behaviour of contaminated PCW materials in deinking and identifies the most effective parameters for ink removal on inhomogeneous flakes.

## 1. Introduction

In 2018, 8.5–9.0 Mt of flexible polyethylene (PE) packaging were produced within the EU28+2, making it the biggest application field for plastics [1]. This is because of their potential to incorporate barriers against oxygen, moisture, and light while assuring high printability, low weight, and low cost [2,3,4,5]. The high production volume of flexible PE packaging, along with their usually short lifespan of a few weeks consequently leads to immense amounts of PCW [6,7]. The recycling of flexible PE packaging poses great challenges, as their exceptional properties are commonly achieved via the introduction of foreign materials and polymers. Multilayer packaging oftentimes contains ethylene vinyl alcohol (EVOH) or even metal barriers, polypropylene (PP) and polyethylene terephthalate (PET) films, and printing inks (generally 2 to 4%) [8], leading to a highly inhomogeneous material flow [7]. While there are barrier technologies, following a mono-material approach [9,10,11], the use of printing inks has not decreased significantly in recent years, as the print serves marketing and mandatory product information purposes [12].

During mechanical recycling, foreign materials can cause degradation and the formation of unwanted side-products. In particular, the degradation products of printing inks, or more specifically their binding agents, are known to be among the main causes of unpleasant odour and colour, as well as a reduction in mechanical properties [7,13,14,15,16,17,18]. Current industrial washing procedures for polymer waste prove unable to remove printing inks from flexible PE waste before regranulation, thus restricting the application range of their recycling material to those with low requirements, such as waste bags or park benches [19]. This conflicts with the EU’s 2030 goals presented in the packaging and packaging waste regulation (PPWR), demanding specific minimum amounts of post-consumer recyclates (PCR) in a variety of packaging solutions, e.g., 10% for contact-sensitive packaging [20]. Considering these developments, the rapid implementation of an ink removal strategy is of great economic and political interest.

In recent years, studies have shown that deinking processes, carried out prior to regranulation, improve recycling quality by minimising the amount of thermally unstable compounds and thus the level of degradation during mechanical recycling [21,22,23]. This deinking process can be explained as washing, oftentimes using high water temperatures and appropriate surfactant and lye formulations. The effectiveness of this process is highly dependent on the film properties (e.g., printing type, lamination, type of binding agent) [24] and the washing parameters (e.g., temperature, pH, surfactant formulation) [14].

Research on model films (oftentimes uniformly surface-printed mono-layer PE films), that allow for straight-forward assessment of ink removal, led to understanding of the deinking mechanism and found generally effective deinking parameters that were recorded to build a methodological basis in the DIN SPEC 91496 in 2024 [5,14,22,23,25,26,27,28,29,30]. As the contamination of PCW films (especially from household collection systems) with, for example, food residues, oils and fats, and other foreign materials can interact with washing solutions and affect deinking, the transferability of effective parameters to PCW flakes should be further investigated to facilitate industrial implementation [2,24].

**Figure 1:**
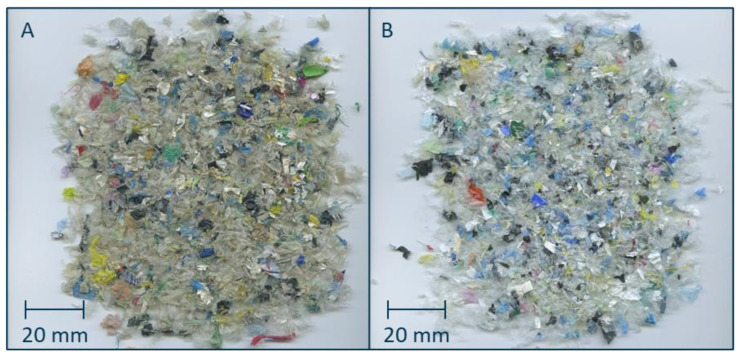
Pictures of PCW flakes prior (**A**) and post (**B**) deinking. Deinking was conducted with a water temperature of 80 °C and a washing time of 120 min.

However, test procedures established for model films cannot be easily implemented for the deinking of PCW flakes. As can be seen in Figure 1, PCW films contain a variety of film structures, from transparent and surface-printed to laminated as well as mass-coloured flakes, that are highly inhomogeneous in colour and type of printing ink. It is therefore necessary to assess a sufficient quantity of flakes with a statistical distribution of said film structures to allow for comparison between batches. Furthermore, the direct comparison of identical flakes before and after deinking is not possible without distorting the composition of the material flow and thus the deinking experiment. Investigating a representative number of flakes before and after deinking (e.g., via scanning electron microscope images or energy dispersive X-ray, as was reported by Gecol et al. [26]) would result in an enormous amount of preparational effort for each deinking experiment.

In recent years, various groups reported quantitative deinking results by measuring L-a-b values according to the standard of the International Commission on Illumination (CIE) [14,26,29,31,32,33,34,35]. Following this procedure, the colour changes of the samples before and after deinking can be calculated using Equation (1) and portrayed as a deinking efficiency using Equation (2).

(1) $$∆E=\sqrt{{∆L}^{2}+{∆a}^{2}+{∆b}^{2}}$$

With:

- ∆E = colour difference of the flakes before and after deinking.
- ∆L = L_experiment_ − L_prior to deinking._
- ∆a = a_experiment_ − a_prior to deinking._
- ∆b = b_experiment_ − b_prior to deinking._

(2) $$DE^{*}=1-\frac{{∆E}_{reference,experiment}}{{∆E}_{reference,prior\;to\;deinking}}\cdot 100\%$$

With:

- ${DE}^{*}$ = deinking efficiency ranging from 0 (no deinking) to 100 (complete deinking).
- $∆E_{\mathrm{reference},\mathrm{sample}}$ = colour difference of the flakes after deinking and transparent reference flakes.
- $∆E_{\mathrm{reference},\mathrm{prior}\;\mathrm{to}\;\mathrm{deinking}}$ = colour difference of the flakes before deinking and transparent reference flakes.

As is evident from the equations, this measurement method requires a reference of transparent films. While this reference is easily obtained for model films, unprinted PCW flakes with identical history can only be obtained via tedious manual sorting of the flakes after deinking. The sorting of either the films before shredding or of the flakes prior to deinking would influence the amount of dissolved printing ink during the experiment and presumably the amount of contamination on the flakes to begin with. It can be assumed that these deviations cause a distortion in the amount of available lye and surfactant molecules. It should also be noted that, due to the existence of non-deinkable flakes in the PCW material flow, deinking rates would be systematically underestimated.

Guo et al. investigated deinking on hand-picked, partially inked LDPE film samples collected from post-consumer sources [31]. Following the CIELab approach, the standard deviations ranged from 11% to 76%, indicating that this method is not suitable for the measurement of PCW flakes. Thus, the flakes were manually sorted into the categories “completely transparent”, “partly transparent” and “not cleaned”, and deinking rate was calculated from the masses of these batches. While this method was proven to be an advancement for the measurement of deinking on surface-printed post-consumer films, the manual sorting of flakes into the abovementioned categories is hardly applicable to real-life household PCW flakes. Furthermore, mass-coloured and laminated flakes cannot be considered as they do not belong in any of the categories.

This study proposes a novel approach for the objective assessment of deinking on household PCW flakes that does not require alterations to or sorting of the waste stream neither before nor after deinking. The aim is to achieve measurable and reproducible results for the deinking of PCW flakes via a straight-forward measurement method and while still following the laboratory-scale approach that was carried out by earlier studies and described in the DIN SPEC 91496 [25]. Via transmitted light scanning, the transparency of the flakes is transformed into the 8-bit grey scale, assigning each pixel a brightness value between 0 and 255. The resulting distributions can then be described and compared by statistical parameters, allowing the objective investigation of the deinking. That being said, the measurement must prove to be robust with respect to the statistical amounts of laminated and mass-coloured flakes as well as the deviations introduced by the folded and rolled-up flakes.

## 2. Materials and Methods

This chapter lists the utilised materials, the sourcing and pre-treatment of the PCW flakes, the deinking procedure, and the evaluation of the ink removal via image-based analysis and statistical characterisation.

### 2.1. Pre-Treatment of PCW Flakes

PCW flakes from the “DSD310” fraction were kindly provided by the Green Dot Holding GmbH & Co. KG, Cologne, Germany. In this fraction, household waste is sorted for flexible PE films. The films are shredded to a size of 40 to 60 mm and separated from coarse impurities and labels using a metal separator, air separator and dry mechanical cleaning. The flakes then undergo a cold washing step including a float–sink separation and are collected before entering regranulation.

To facilitate handling and increase the specific surface area for the following deinking trials, the flakes were dried at 80 °C for 6 h and cut to a target size of 10–20 mm using a MDSi 410/200 cutting mill from Hellweg Maschinenbau GmbH & Co. KG, Roetgen, Germany.

### 2.2. Deinking Trials

The washing trials were based on the DIN SPEC 91496 but were modified to account for the peculiarities of PCW flakes [25]. The water temperature varied in three steps (30 °C, 60 °C, and 80 °C), with the lowest temperature marking a change compared to the 40 °C proposed in the DIN SPEC 91496. This change was made to represent the temperature of a cold washing process as is oftentimes used in current industrial washing processes. In contrast to the DIN SPEC 91496, which intends the removal of a single flake sample at specific times in a period of 15 min, trials in this study were conducted at three different washing times of 15, 60 and 120 min without the removal of flake samples [25]. This is because the removal of a single PCW flake does not reflect the deinking of the highly inhomogeneous fraction. Furthermore, washing times were significantly increased compared to the DIN SPEC 91496 [25]. This change accounts for the contamination on PCW flakes that interact with the washing solution, potentially reducing their deinking capability. Moreover, the increased washing times should lead to increased friction on the flakes, further promoting the penetration of the washing solution and thus ink removal. As dissolved printing inks can recolour the flakes via migration into the polymer matrix, an effect that would increase with increased washing time, a transparent virgin PE flake was added to each experiment to trace possible recolouring of the flakes. However, none of the trials showed any sign of recolouring, which is most likely due to the overall lower concentration of printing inks in PCW flake fractions compared to the model films used in other studies. Lye as well as surfactant concentration were kept as in the DIN SPEC 91496 at 1% and 0.2% respectively [25]. Accounting for the inhomogeneity of PCW flakes, the general setup was increased to wash 10 g of flakes per trial. In a 2 L beaker, the surfactant (1-Hexadecyl)trimethylammonium chloride (96%), also known as Cetronium chloride (CTAC, supplied from Thermo Fischer Scientific, Darmstadt, Germany) was dissolved in a solution (1 L) of sodium hydroxide (≥98%, supplied from Carl Roth GmbH + Co. KG, Karlsruhe, Germany), and the mixture was held at the target temperature. PCW flakes were stirred in the solution for 200 min^−1^ for the target washing time. The flakes were then removed from the washing solution, rinsed with 0.5 L of water and dried in a convection oven at 80 °C for 6 h.

### 2.3. Evaluation of Ink Removal

Transmission measurements are conducted on a Perfection V750 Pro from SEIKO Epson Corporation, Nagano, Japan. For the measurement, a 2 g batch of flakes is placed on the scanning surface and manually distributed to ensure an even distribution of flakes without them overlapping. This measurement is repeated four times for each experiment, with the next 2 g batch respectively, yielding a total of four scans and 8 g of scanned flakes per experiment. Scans are then transformed into greyscale and the grey values of all pixels below a threshold of 240 are evaluated. This threshold ensures that both the background, which due to total transparency, has the maximum grey value of 255, and distorting light effects causing higher grey values are excluded from the evaluation while still ensuring the complete incorporation of transparent flakes. Grey values from all scans of an experiment are further processed into an experiment’s distribution with the aim of achieving reproducible results despite the existence of mass-coloured and laminated flakes as well as process-related wrinkles of the flakes. From these distributions, normalised histograms and statistical characteristics (skewness, median, and quartiles) can be calculated, allowing a quantitative comparison between experiments.

### 2.4. Calculation of Statistical Characteristics

**Figure 2:**
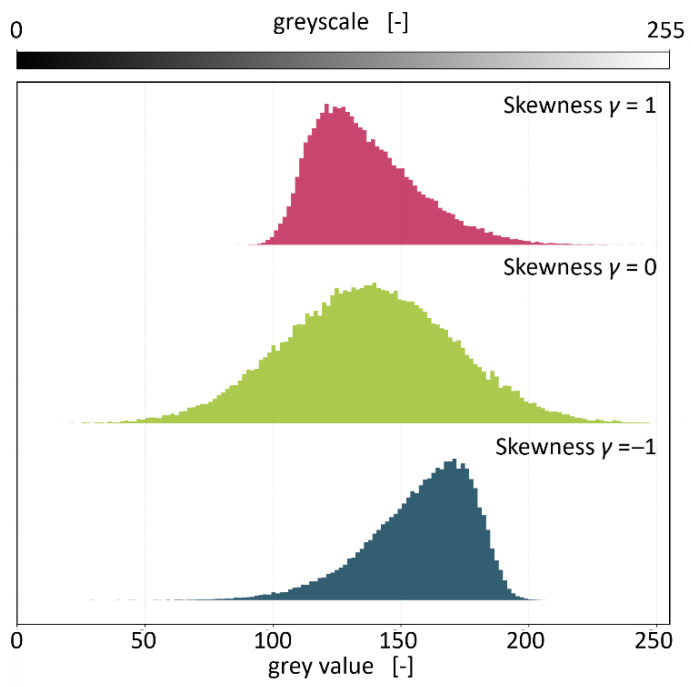
Exemplary grey value distributions and their respective skewness.

The characteristics used for the evaluation in this study are quartiles and skewness. The quartiles *Q*_1_, *Q*_2_ (also called median), and *Q*_3_ divide the grey value distributions into four parts of equal size. Hence, 25% of the grey values lie below *Q*_1_, half of the grey values lie below *Q*_2_, and 75% of the grey values lie below *Q*_3_, respectively. These values are the basis for the generation of box plots [36,37]. The skewness is a measure to describe the symmetry of a distribution. Skewness is mathematically defined as the third central moment normalised by the standard deviation and is derived in Equations (3)–(6) below [36,37,38]. A ridge plot, depicting exemplary distributions, is shown in Figure 2.

(3) $$mean\;grey\;value\;\mu =\frac{1}{N}\sum_{i}x_{i}h_{i}$$

With:

- $x_{i}$ = grey values between 0 and 240.
- $h_{i}$ = number of pixels with a certain grey value.
- N = total pixel count $\left(N=\;\sum_{i}h_{i}\right)$.

(4) $$variance\;\sigma^{2}=\frac{1}{N}\sum_{i}{{(x}_{i}-\mu )}^{2}h_{i}$$

With:

- σ = standard deviation $\left(\sigma =\;\sqrt{\sigma^{2}}\right)$

(5) $$third\;central\;moment\;m_{3}=\frac{1}{N}\sum_{i}{{(x}_{i}-\mu )}^{3}h_{i}$$

(6) $$Skewness\;\gamma =\frac{m_{3}}{\sigma^{3}}$$

As can be seen in Figure 2, the skewness is equal to zero for normal distributions. A positive skew indicates that the mass of the distribution is concentrated to the left side of the distribution, in this case towards lower grey values, with tailing to the right side of the distribution. Consequently, a negative skew indicates that the mass of the distribution is concentrated to the right side of the distribution with tailing to the left side of the distribution. In the context of deinking on PCW flakes, a shift in grey values towards higher values, thus higher transparency, is expected. Therefore, the skewness can be used as a measure to evaluate the effectiveness of deinking experiments. It should however be noted that a skew of zero is also possible if one tail of the distribution is long and thin, while the other is short and fat. Hence, the skewness should not be used as a single evaluation metric without inspecting the shape of the distribution.

## 3. Results and Discussion

### 3.1. Determination of the Grey Value Threshold

**Figure 3:**
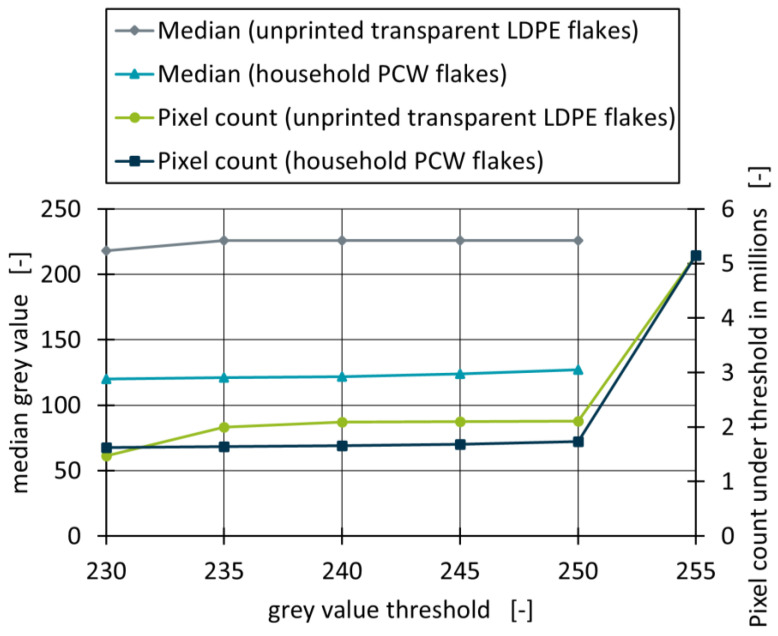
Median grey values and pixel count under the respective grey value thresholds for both unprinted transparent LDPE flakes (without wrinkles) and household PCW flakes.

As described in Section 2.3, a grey value threshold is required to exclude the background of the scanned samples as well as light reflections while still entirely including transparent flakes. The optimal threshold was determined by scanning both unprinted transparent LDPE flakes and PCW flakes, setting the grey value threshold to 230, 235, 240, 245, and 250, and calculating the number of pixels as well as the median grey value under the respective thresholds, which are depicted in Figure 3. Scans with the respective thresholds are also visualised as heatmaps (see Appendix A).

Unprinted transparent LDPE flakes that laid flat on the scanner surface were defined as the upper limit that must be completely included in the measurement method. At a grey value threshold of 230, the flake median amounts to 218. With increasing threshold, the median increases to a constant value of 226. Removal of the threshold consequently increases the median to the maximum grey value of 255, since the scanning background is no longer removed. Pixel counts for the unprinted transparent flakes amount to 1.47 million at a threshold of 230 and increase to 2.0 million pixels at a threshold of 235. At the threshold of 240, 245, and 250, 2.1 million pixels are measured, which increase to the total amount of 5.1 million pixels that are measured on the scanned surface when no threshold is set. Considering these shifts in median and pixel count as well as the heatmaps, it can be concluded that the threshold should be between 235 and 250 to fully include the pixels measured from unprinted transparent LDPE flakes. With increasing grey value thresholds, median grey values amount to 120, 121, 122, 124, and 127, respectively. Pixel counts amount to 1.6 million for the thresholds of 230 and 235, and to 1.7 million for 240, 245 and 250. In the case of PCW flakes, the slight but steady shift in the median grey value indicates that light reflections on small particles and flake edges (both of which are predominantly caused by the shredding of the films during mechanical recycling) are increasingly included in the calculations when increasing the grey value threshold. This effect can also be observed in the respective heatmaps. For both materials, mostly constant median grey values and pixel numbers could be achieved at a grey value threshold of 240, which is why it was chosen as the threshold for subsequent investigations.

### 3.2. Inivestigation of the Method Robustness

**Figure 4:**
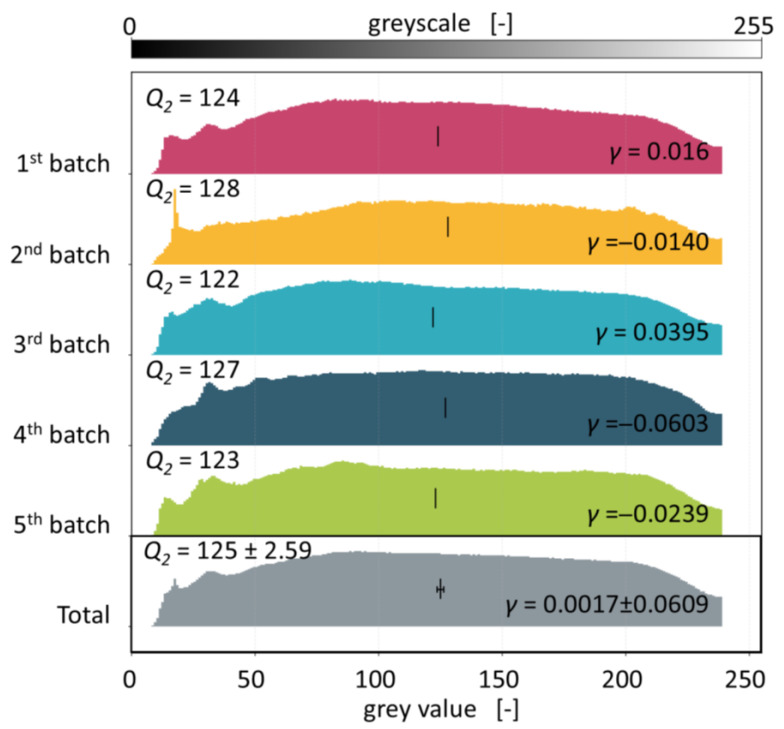
Ridge plot of five batches of PCW flakes prior to deinking and total normalised histograms containing the grey values of all scans. Skewnesses *γ* and median grey values *Q*_2_ (depicted by the vertical stroke in each histogram) are given for all batches.

To gain an insight into the reproducibility and robustness of the aforementioned evaluation method, the scanning routine (four scans of 2 g of PCW flakes respectively) was repeated five times, yielding a total of 20 scans and 40 g of scanned flakes. Comparison of these results incorporate not only the inaccuracies of the measurement practice (e.g., wrinkles and overlapping of the flakes) but also the inhomogeneity of the PCW fraction (laminated and mass-coloured flakes). Hence, the standard deviations on the skewness and median of this experiment were adopted for all further deinked experiments. A ridge plot depicting the normalised histograms of the five flake batches as well as the total normalised histogram, containing the grey values of all batches, is shown in Figure 4.

The normalised histograms depicted in Figure 4 are similar in shape and almost normally distributed, which is confirmed by the skewness close to 0. Peaks in the grey value range of 15 to 20 and 30 to 40 are prominent in some of the histograms. These peaks can be assigned to mass-coloured and laminated flakes that are statistically distributed in the investigated PCW fraction. In particular, black recycling flakes, originating from waste bags that are currently produced from the material of this PCW fraction, have grey values of around 20 (see Appendix B). Batches in which these flakes are disproportionately included show a sharp peak in this area, as can be seen in batch 2. Considering the obvious fluctuations in the amount of mass-coloured and laminated flakes, the total skewness amounts to 0.0017 with a standard deviation of 0.0609 and the median grey value amounts to 125 with a standard deviation of 2.59. As these deviations include the background noise introduced by the inhomogeneity of the material flow as well as by the flakes’ overlaps and wrinkles, they will subsequently be used as a measure of evaluation for the significance and effectiveness of the deinking experiments.

**Figure 5:**
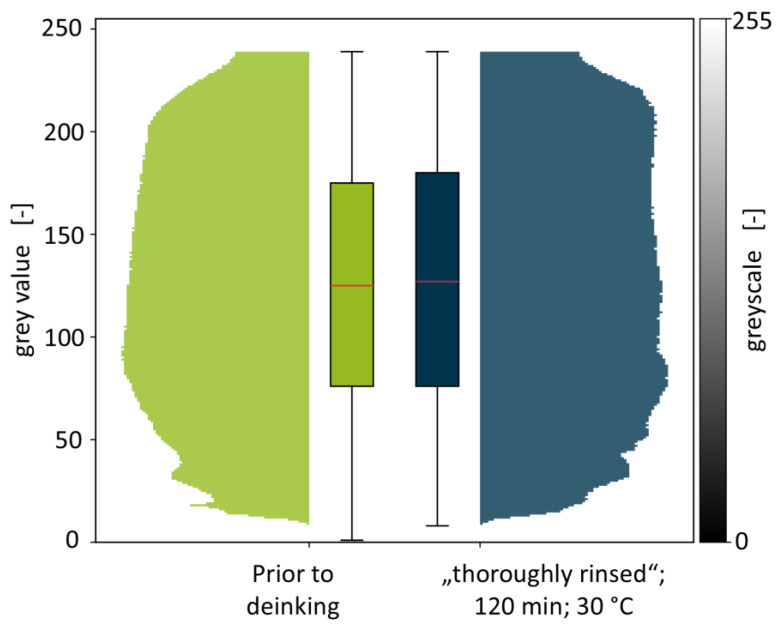
Violin plot for the comparison of the PCW flakes prior to deinking and after stirring for 120 min at 30 °C, followed by thorough rinsing. Box plots of the respective experiments are shown to further describe the grey value distributions.

Although the PCW flakes investigated in this study have undergone an industrial cold washing procedure, as was mentioned in Section 2.1, residues of contaminants cannot be ruled out. Consequently, a shift towards higher grey values that is achieved in deinking experiments could also be due to removal of contaminants instead of printing inks. The extent of this effect was investigated by stirring a 10 g batch of the PCW flakes in water at 30 °C for 120 min, followed by thoroughly rinsing the flakes under flowing water. This should remove most remaining contaminants while leaving the printing ink layers intact. A direct comparison of PCW flakes before deinking and after thorough rinsing is depicted in Figure 5.

PCW flakes prior to deinking, as already mentioned above, show a near-normal distribution of grey values with a skewness of 0.0017 ± 0.609 and a median grey value of 125 ± 2.59. The histogram of the PCW flakes that underwent additional cold washing is similarly distributed. The skewness for this experiment amounts to −0.0043 with 127 being the median grey value. It can be concluded that both the skewness and the median grey value of the flakes after additional cold washing is well within the range of the PCW flake batches shown in Figure 4. While this experiment cannot serve as proof that residual contamination has no influence on the measured grey values, it indicates that contaminant residues have a negligible effect on the grey value distributions for the PCW flakes that are investigated in this study. Consequently, a significant shift in grey values for deinking trials can be considered a strong indicator for the removal of printing inks.

### 3.3. Deinking Trials Under Variation in the Water Temperature and Washing Time

**Figure 6:**
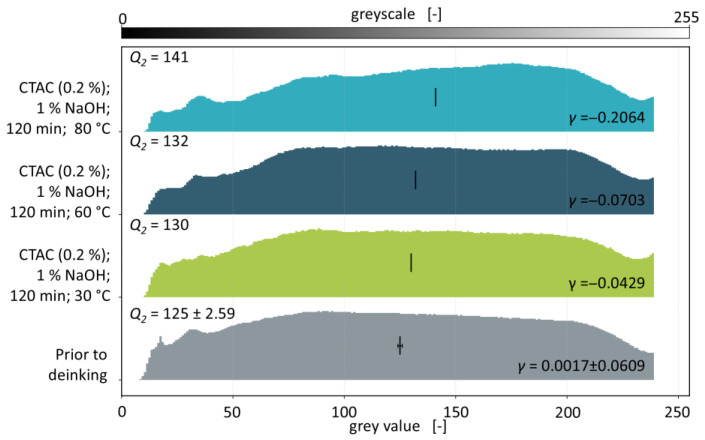
Ridge plot containing normalised grey value histograms of PCW flakes prior and post deinking with varying water temperatures. Skewnesses *γ* and median grey values *Q*_2_ (depicted by the vertical stroke in each histogram) are given for all experiments.

Deinking experiments were based on the parameters of the DIN SPEC 91496 with alterations made to account for the peculiarities of PCW flakes [25]. Trials using 1% NaOH and 0.2% CTAC were varied in washing time and water temperature, whereby an increase in those parameters is expected to result in an increase in the deinking performance. A picture of the flakes prior (A) and post (B) deinking with a water temperature of 80 °C and a washing time of 120 min is shown in Figure 6.

While the general brightening of the flakes due to deinking is apparent (see Figure 1), mass-coloured and laminated flakes are not affected by deinking and thus lead to a mixed appearance that cannot be quantified visually. The ridge plots of the deinking experiments with a washing time of 120 min and varying water temperatures are shown in Figure 6.

The histograms of deinked batches depicted in Figure 6 show a slight shift towards higher grey values compared to the batch prior to deinking, indicating a higher transparency and thus the removal of printing inks from the PCW flakes. Especially at a temperature of 80 °C, the number of pixels below a grey value of 100 is decreased, while the amount in the range of 150 to 200 is increased. This observation is confirmed quantitatively by the skewnesses that decrease with increasing washing temperature, marking a reduction in the skewness of 0.0446 at 30 °C, 0.0720 at 60 °C, and 0.2081 at 80 °C respectively. Median grey values increase from 125 ± 2.59 prior to deinking to 130 at 30 °C, 132 at 60 °C, and 141 at 80 °C. With increasing water temperatures, median grey values increase by 5, 7, and 16 respectively.

**Figure 7:**
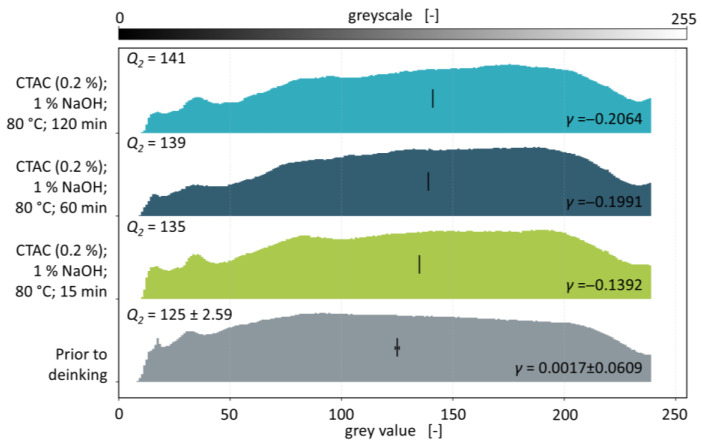
Ridge plot containing normalised grey value histograms of PCW flakes prior and post deinking with varying washing times. Skewnesses *γ* and median grey values *Q*_2_ (depicted by the vertical stroke in each histogram) are given for all experiments.

Considering the deviations on the skewness and median grey value of the batch prior to deinking, a significant decrease in the skewness is achieved only for the experiment at a water temperature of 80 °C. At higher temperatures, the diffusion of surfactants and lye into the printing ink layers is accelerated and the layer itself expands and softens, further promoting the diffusion. These results are in accordance with the studies on model films published to date and thus indicate that the measurement method is capable of detecting differences in the deinking efficiency for varying washing parameters. Keeping the most efficient water temperature of 80 °C, deinking experiments with different washing times are depicted in Figure 7.

The histograms of the deinked batches depicted in Figure 7 show a shift towards higher grey values compared to the sample prior to deinking. This again indicates higher flake transparency and thus the removal of printing inks. A decrease in the number of pixels below a grey value of 100 as well as an increase in the amount in the range of 150 to 200 can be observed for all washing times. Quantifying this observation, the decrease in the skewnesses amounts to 0.1409 (83%) after 15 min, 0.2008 (118%) after 60 min, and 0.2081 (122%) after 120 min respectively. Median grey values increase from 125 ± 2.59 prior to deinking to 135 after 15 min, 139 after 60 min, and 141 after 120 min of washing. Consequently, median grey values increase by 10, 14, and 16 with increasing washing times.

**Figure 8:**
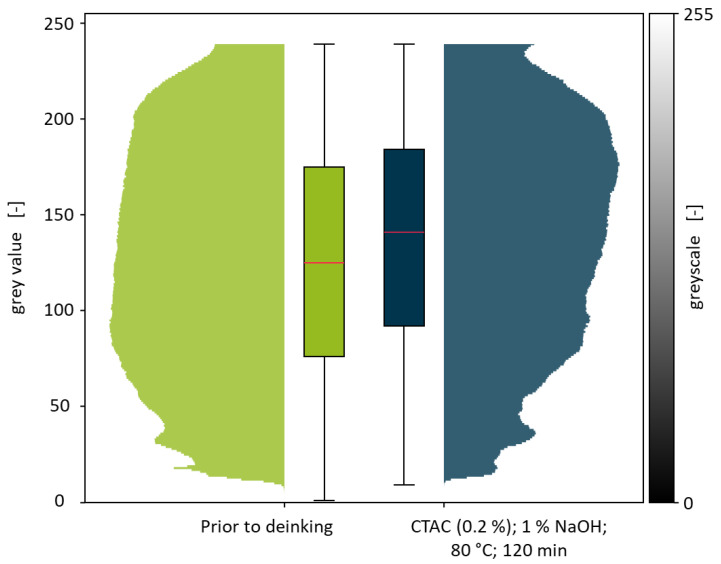
Violin plot for the comparison of the PCW flake batches prior to deinking and the batch deinked with a water temperature of 80 °C and a washing time of 120 min. Box plots of the respective experiments are shown to further describe the grey value distributions.

A significant change in skewness and median with respect to the flakes prior to deinking can be shown for all experiments, with only minor changes for the experiments with a washing temperature of 60 and 120 min. These results indicate that the dissolution of printing inks is still in progress after 15 min but could be duly completed by 60 min. In the DIN SPEC 91496, the maximum washing time is set to 15 min [25]. This is because the deinking of surface-printed films usually proceeds rapidly under the proposed conditions as the ink layer is readily accessible to the washing solution. Furthermore, recolouring is oftentimes observed in the deinking of these films which renders unnecessary high washing times counterproductive. PCW fractions, being composed of surface- and reverse-printed but also mass-coloured, laminated and transparent flakes, usually possess lower amounts of dissolvable printing ink than model films. Hence, no recolouring could be observed in any of the deinking experiments using PCW flakes. The increased washing times are meant to account for the contamination residues on the flakes and to introduce friction via the prolonged wiping of the flakes onto each other. However, the effect of this friction cannot be investigated in the course of these experiments and is expected to be negligible compared to the friction that is introduced via state-of-the-art polymer washing devices that are currently investigated in the pilot scale [39]. A direct comparison of the batch prior to deinking with the deinked batch using a water temperature of 80 °C and a washing time of 120 min is shown in Figure 8.

The direct comparison of the PCW flake batches prior and post deinking in a violin plot further visualises the shift in grey values that is caused by the deinking process. While the pixel count significantly decreases at grey values below 100, it increases for grey values above 150. In addition to the shifts in the median grey values and skewness already discussed, box plots show an increase in the first and third quantiles *Q*_1_ and *Q*_3_. *Q*_1_ increased from 76 to 92, marking an increase of 16. *Q*_3_ increases from 175 to 184, which is a grey value increase of 9. It can thus be concluded that both the skewness and the quantiles can be used to describe the effectiveness of deinking trials. The whiskers of the box plot however cannot display the interquartile range for these broad distributions, which is why they are defined as the minimum and maximum grey value measured for the experiment.

### 3.4. Assessment of the Measurement Methodology

**Figure 9:**
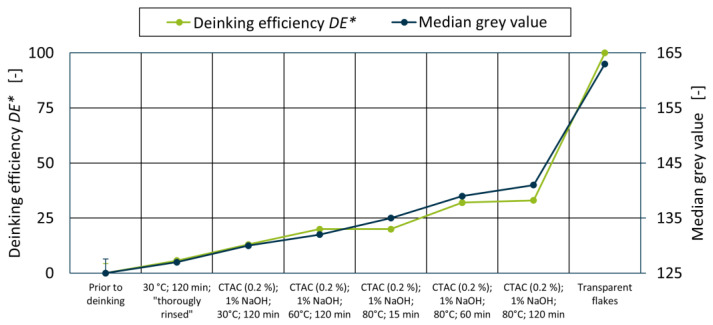
Deinking efficiencies DE* and median grey values for the respective deinking experiments.

Putting these results into perspective, deinking efficiencies DE* were calculated after Equations (1) and (2) to allow for a comparison of the presented evaluation method to the CIELab method that is established for the evaluation of deinking experiments on model films. As this method requires an unprinted reference, transparent flakes were sorted manually from a deinked experiment to serve as a reference without printing ink, but with wrinkles and folded flakes. A comparison of the median grey values and deinking efficiencies measured by the CIELab method is shown in Figure 9.

As can be seen from Figure 9, both evaluation methods indicate better deinking with increased washing time and water temperature, with a similar sensitivity towards both parameters. However, the established definition of the deinking efficiency, with zero indicating that no deinking has taken place and 100 indicating complete ink removal, cannot be applied to this material. The existence of laminated and mass-coloured flakes leads to a systematic underestimation of the actual ink removal for all trials. It should also be noted that the introduction of a transparent reference introduces an additional error to this measurement method, which was neglected for this direct comparison. When investigating materials from another source, additional references would be required, further increasing the preparatory effort of the CIELab method. Other than that, the CIELab method inevitably shares all measurement uncertainties with the grey value method, as both methods must overcome the process-related inhomogeneities of the PCW flakes.

From a practical point of view, the proposed measurement method offers a simpler evaluation of deinking experiments, eliminating the need for reference samples while showing reproducibility and robustness comparable to the CIELab method. Grey value distributions enable the calculation of statistical characteristics and thus objective comparison of deinking experiments. Expanding on the deinking experiments by further varying the parameters investigated in this study, but also the lye and surfactant concentration as well as the surfactant formulations, would enable the investigation of correlations between the deinking parameters and thus provide additional insight into the deinking mechanism.

## 4. Conclusions

This study proposes a novel measurement method to objectively quantify the deinking efficiency of household PCW flakes. Deinking trials were based on the washing parameters listed in the DIN SPEC 91496 with few alterations made to account for the peculiarities of PCW flakes. The deinking setup was scaled to five times the size, with 10 g of PCW flakes being washed in a beaker of 2 L, to minimise the effect of the statistically distributed laminated and mass-coloured flakes while still allowing for a straight-forward lab-scale experiment design.

It could be shown that the image-based analysis method, using grey values to create histograms and comparing skewnesses as well as median grey values, yields sufficiently accurate results to allow for the observation of significant changes in the experiments. Trends, that were previously confirmed by other researchers, could be observed and quantified without manual sorting of the inhomogeneous material flow. With an increase in the grey value median of 17, the deinking trial with the highest water temperature of 80 °C and the longest washing time of 120 min showed the highest deinking effect for the experiments investigated in this study. Within the range of the parameters investigated, the water temperature showed to have a greater effect on the deinking efficiency than the washing time. It should be noted that for industrial-scale deinking experiments under the incorporation of friction, deinking would be accelerated, thus achieving significant ink removal in a fraction of the washing times presented in this study [39].

Additional deinking experiments, expanding the parameters to various surfactants, lye concentrations, film structures, and deinking setups, would put the method further to test, allowing improvements to the analysis method. Further variations in the washing time and water temperature would enable the quantification of their correlation with deinking efficiency.

## Figures and Tables

**Figure A1:**
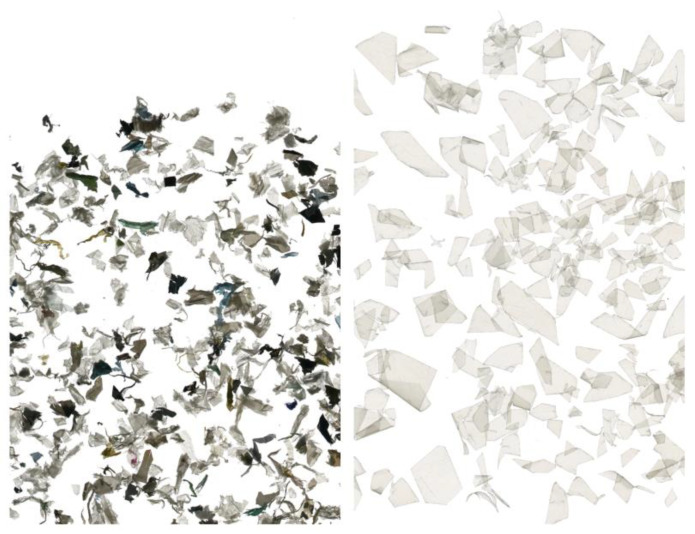
Scans of household PCW flakes prior to deinking that were used in this study (**left**) and unprinted transparent LDPE flakes (**right**).

**Figure A2:**
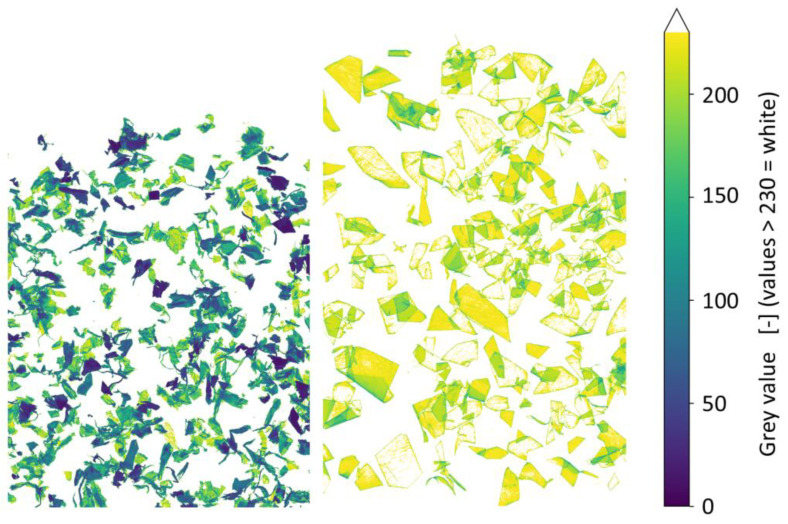
Scans of household PCW flakes prior to deinking (**left**) and unprinted transparent LDPE flakes (**right**) depicted as heatmaps with 230 as the grey value threshold. At this threshold, pixels from the transparent flakes are partially excluded from the evaluation.

**Figure A3:**
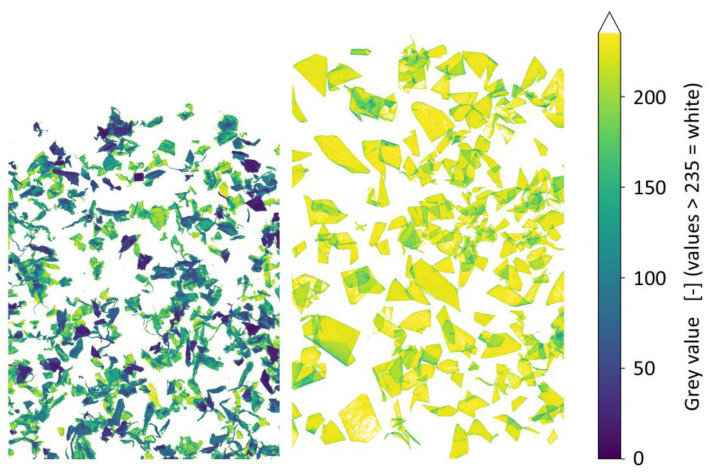
Scans of household PCW flakes prior to deinking (**left**) and unprinted transparent LDPE flakes (**right**) depicted as heatmaps with 235 as the grey value threshold. At this threshold, few pixels from the transparent flakes are excluded from the evaluation.

**Figure A4:**
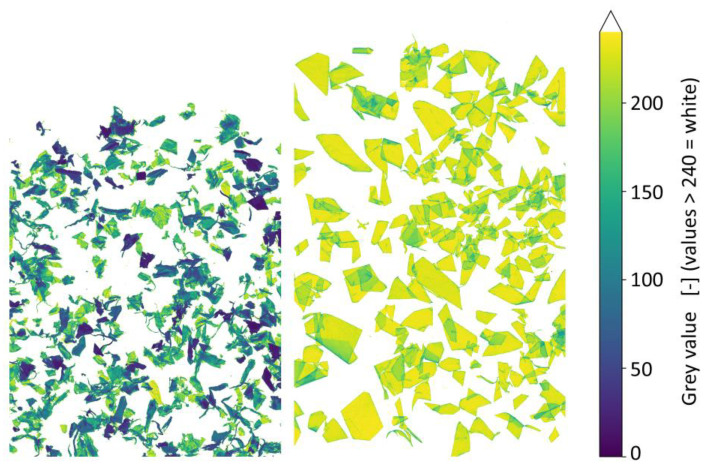
Scans of household PCW flakes prior to deinking (**left**) and unprinted transparent LDPE flakes (**right**) depicted as heatmaps with 240 as the grey value threshold. At this threshold, pixels from the transparent flakes are fully included in the evaluation and only a few small particles are visible in the PCW flake batch.

**Figure A5:**
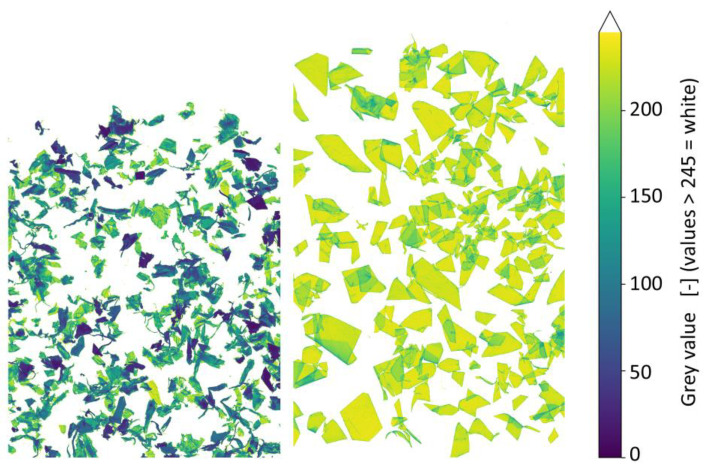
Scans of household PCW flakes prior to deinking (**left**) and unprinted transparent LDPE flakes (**right**) depicted as heatmaps with 245 as the grey value threshold. At this threshold, pixels from the transparent flakes are fully included in the evaluation and only a few small particles are visible in the PCW flake batch. Slight brightening on the flake edges can be seen, indicating reflections on the ragged edges of the PCW flakes.

**Figure A6:**
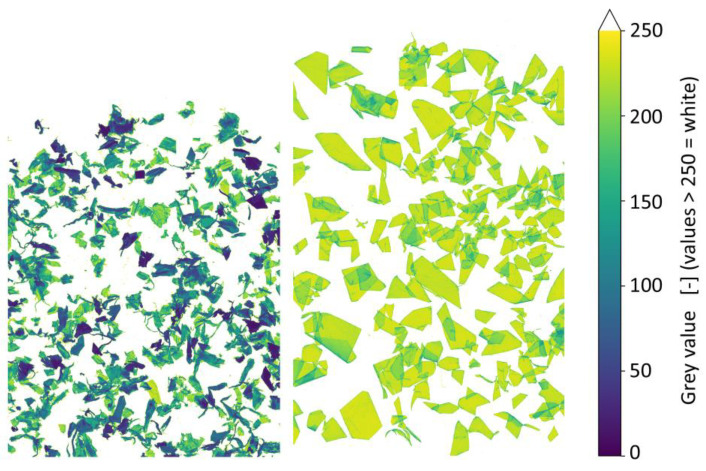
Scans of household PCW flakes prior to deinking (**left**) and unprinted transparent LDPE flakes (**right**) depicted as heatmaps with 250 as the grey value threshold. At this threshold, pixels from the transparent flakes are fully included in the evaluation and some small particles are visible in the PCW flake batch. Slight brightening on the flake edges can be seen, indicating reflections on the ragged edges of the PCW flakes.

**Figure A7:**
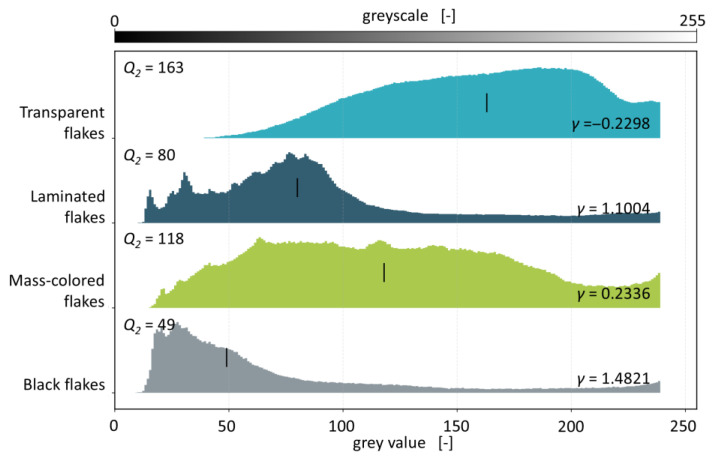
Ridge plot containing normalised grey value histograms of manually sorted fractions of PCW flakes after deinking. Skewnesses γ and median grey values *Q*_2_ (depicted by the vertical stroke in each histogram) are given for all experiments.

## Data Availability

All data presented in this study are available on request from the corresponding author.

## Abbreviations

The following abbreviations are used in this manuscript:

PE	Polyethylene
CIE	International Commission on Illumination
LDPE	Low-density polyethylene
PCW	Post-consumer waste
EVOH	Ethylene vinyl alcohol
PP	Polypropylene
PET	Polyethylene terephthalate
PPWR	Packaging and packaging waste regulation
PCR	Post-consumer recyclate
*DE**	Deinking efficiency
CTAC	Cetronium chloride

## Appendix B

Flakes of a deinked experiment were manually sorted for the categories “black”, “mass-coloured”, “laminated”, and “transparent”. Separate scans of these film structures allow additional insight into the measurement method as they allow a rough classification of film structures and their corresponding grey value distributions. The ridge plot is depicted in Figure A7.

As can be seen from the normalised histograms depicted in Figure A7, manual sorting of PCW flakes results in clear differences in the fractions’ grey value distributions. Black flakes, that mostly originate from recycling materials like waste bags, were sorted individually from other mass-coloured flakes. The histogram shows a shift towards lower grey values, with a median grey value of 49 and most pixels being below a grey value of 60. Other mass-coloured flakes include blue, green, orange, purple, pink, red, and yellow flakes and consequently show a broad grey value distribution with a median grey value of 118. As these flakes originate from various sources and differ significantly in both film thickness and colour intensity, this was to be expected. While black flakes also differ in film thickness, they oftentimes contain carbon, rendering them more opaque than other mass-coloured flakes. Laminated flakes show multiple peaks below grey values of 100 and a median grey value of 80. Again, this fraction contains flakes of various sources, varying in film thickness and film design. Laminated films containing a metal barrier of darker printing layers lead to peaks in the lower grey value range. Finally, the normalised histogram of the transparent flake fraction is shifted towards higher grey values with a median grey value of 163. The tailing towards lower grey values is due to flakes overlapping and folding, leading to lower transparency. However, as it is difficult to distinguish between mass-coloured white flakes and transparent flakes, the sorting quality of this fraction should be carefully considered. Furthermore, the histograms shown should not be taken as representative of said flake fractions. Due to the tedious manual sorting of the flakes after deinking, the total mass of the fractions used for this analysis amounts to 1 g.
